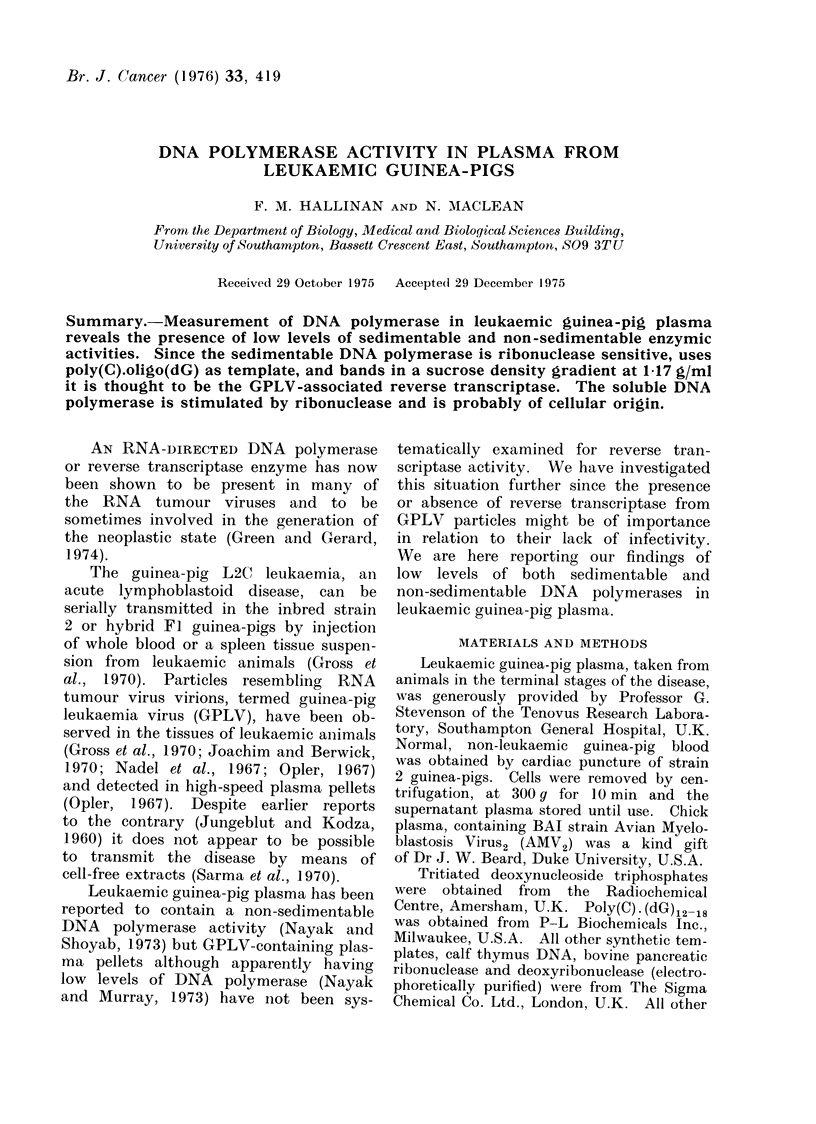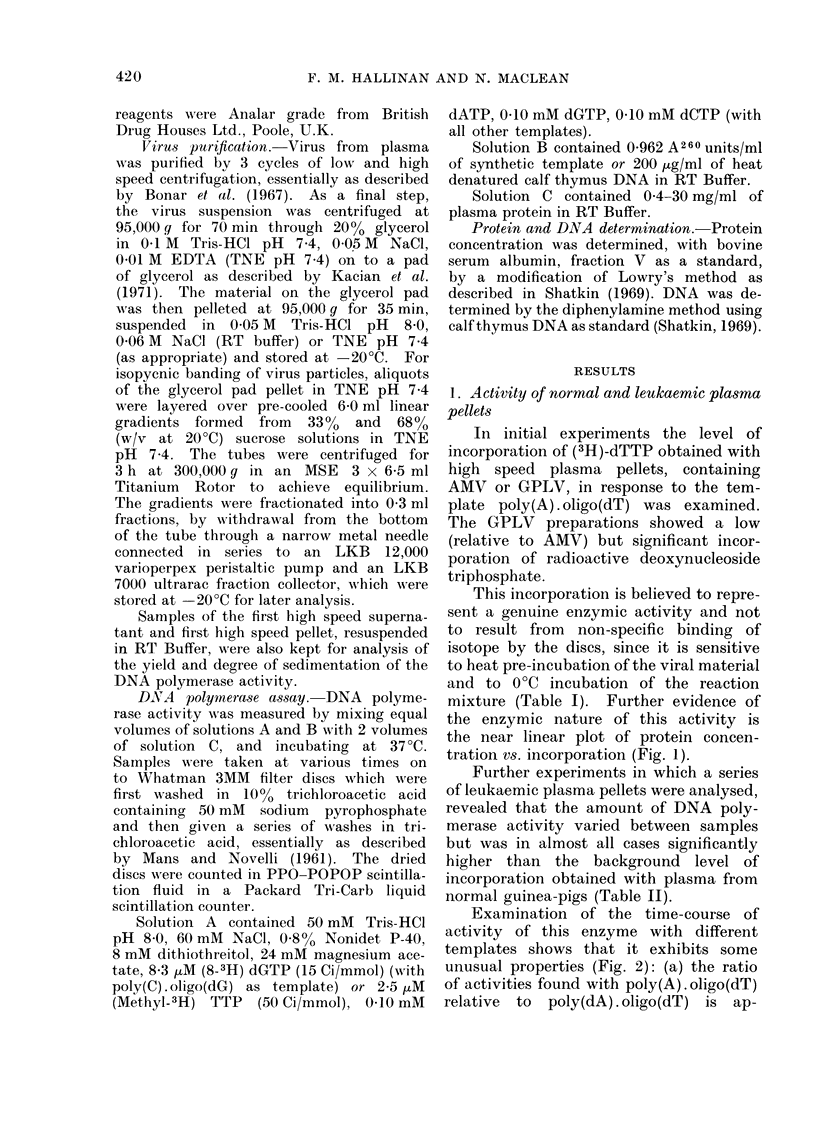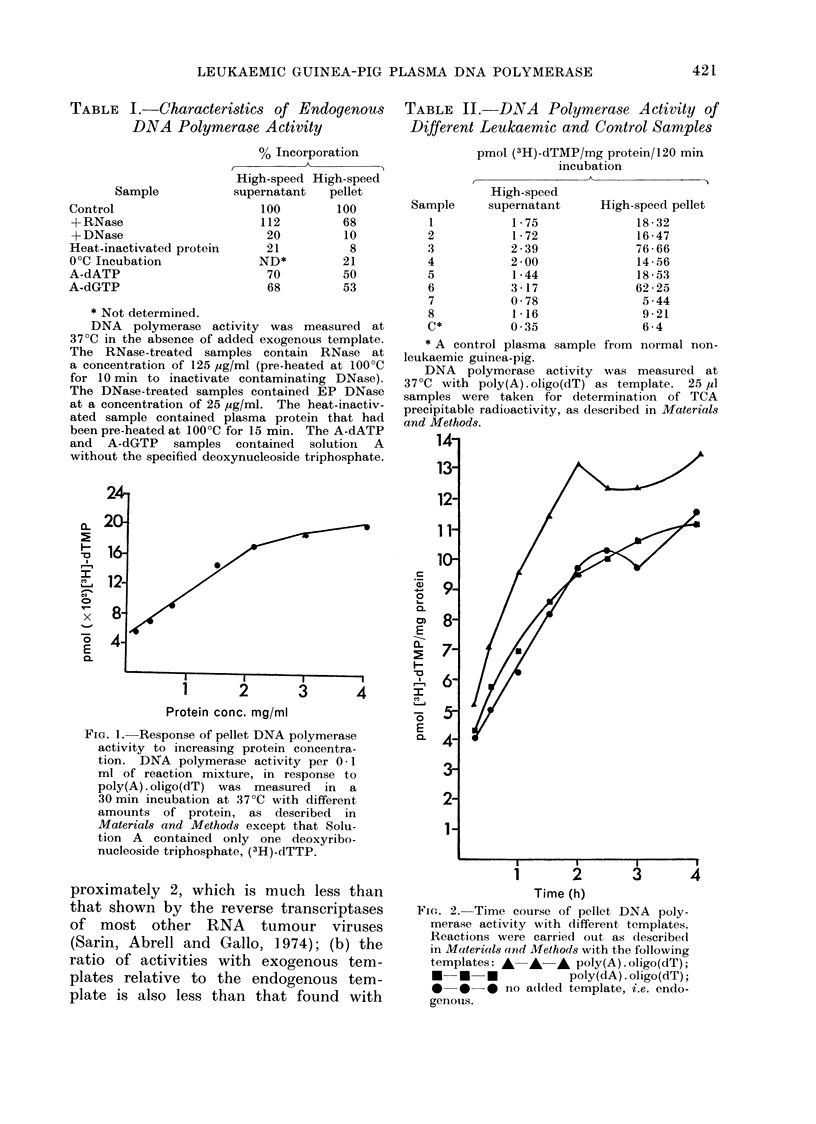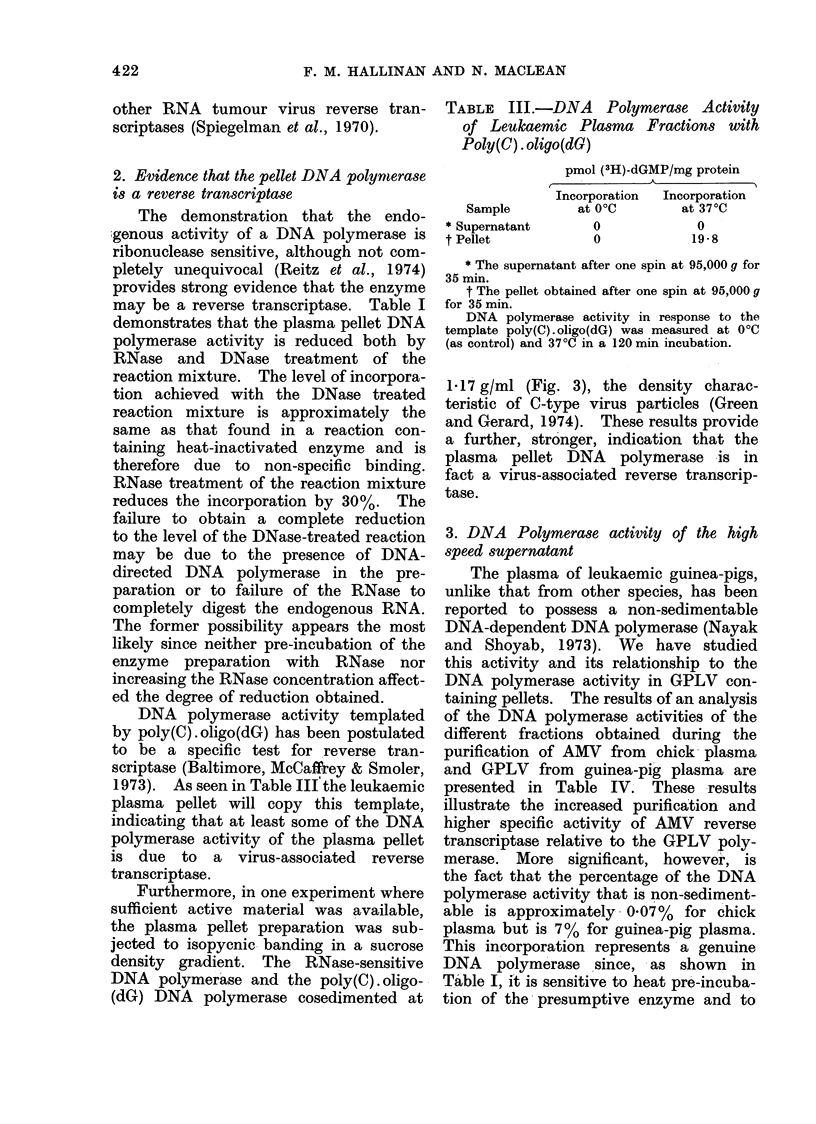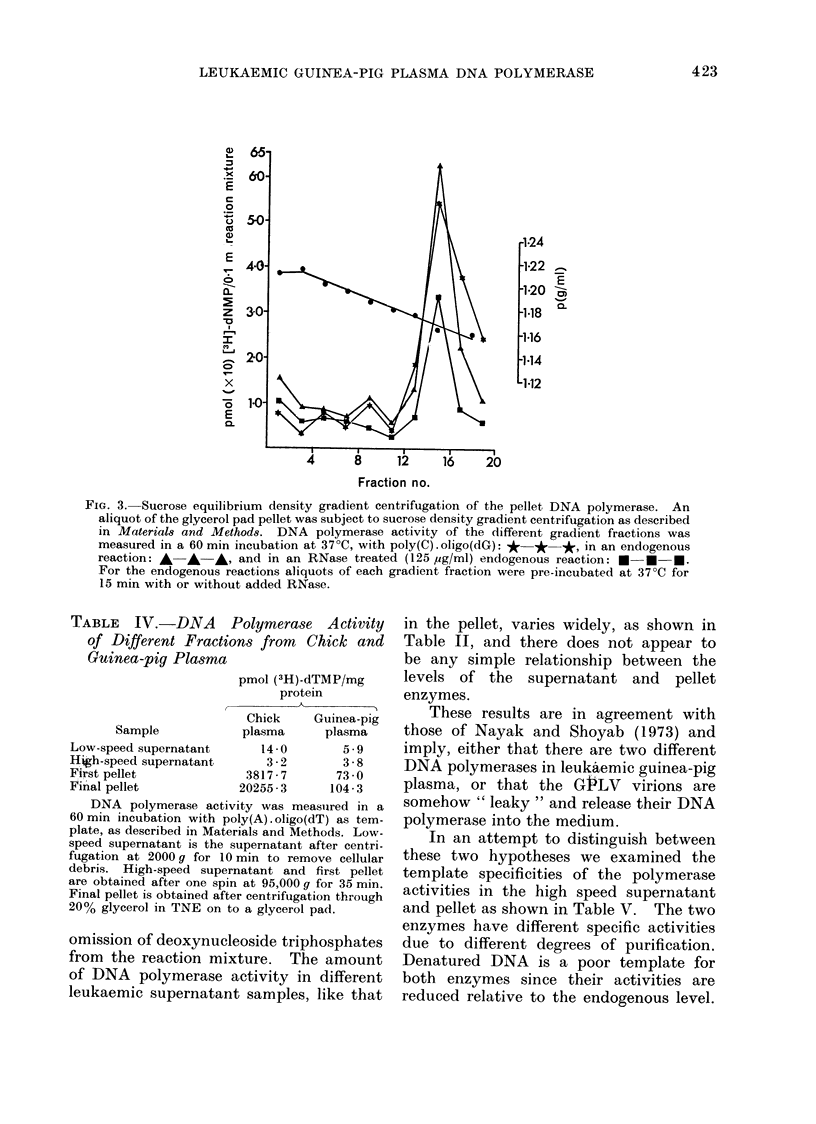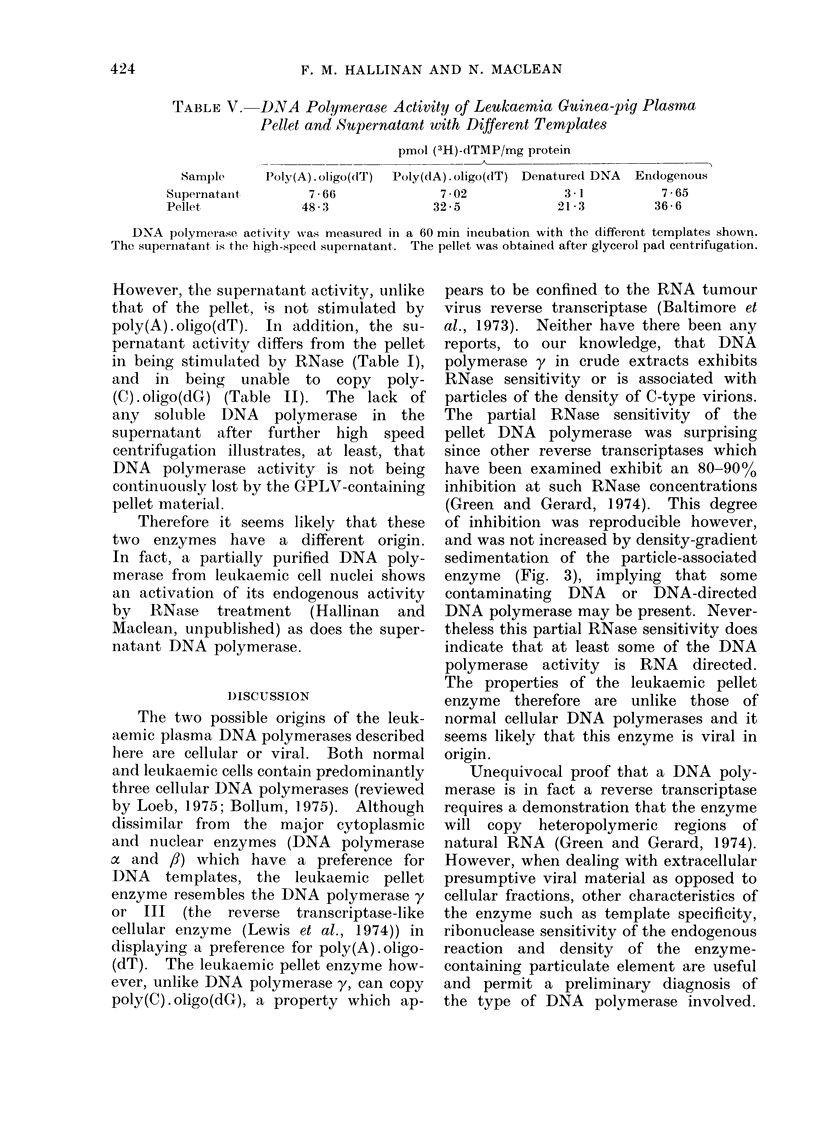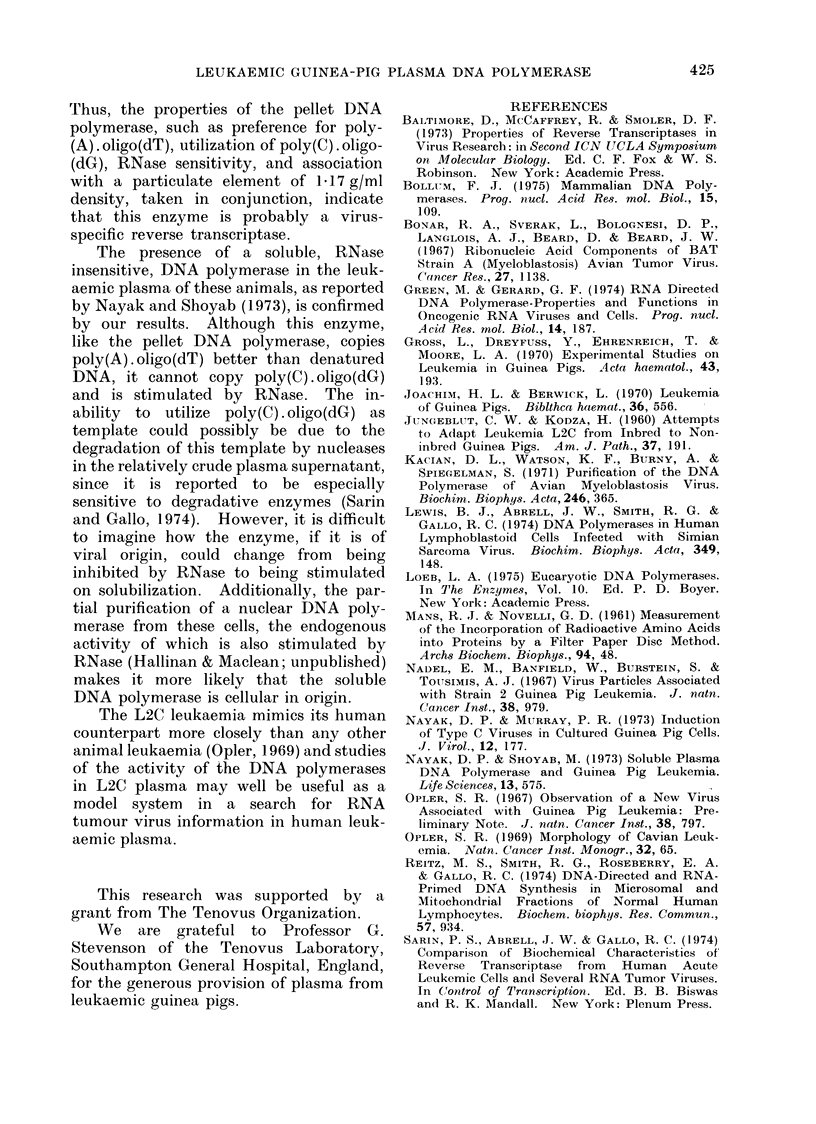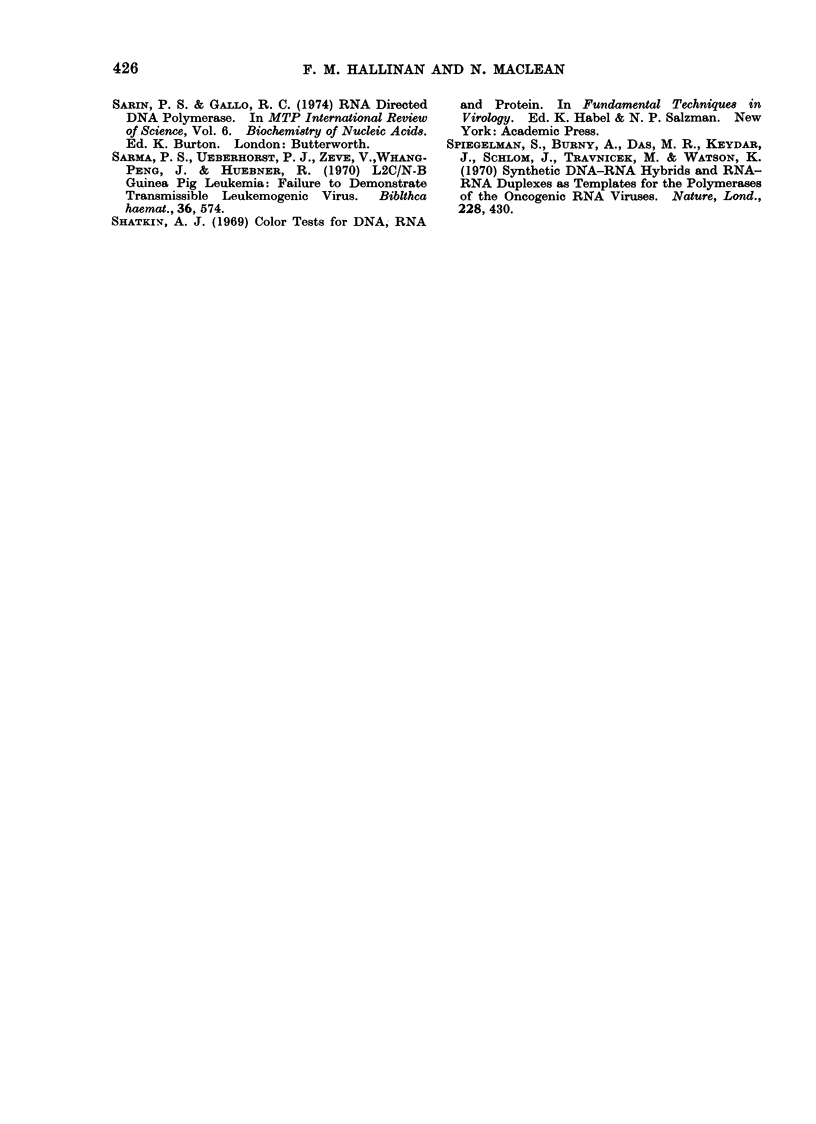# DNA polymerase activity in plasma from leukaemic guinea-pigs.

**DOI:** 10.1038/bjc.1976.67

**Published:** 1976-04

**Authors:** F. M. Hallinan, N. Maclean

## Abstract

Measurement of DNA polymerase in leukaemic guinea-pig plasms reveals the presence of low levels of sedimentable and non-sedimentable enzymic activities. Since the sedimentable DNA polymerase is ribonuclease sensitive, uses poly(C).oligo(dG) as template, and bands in a sucrose density gradient at 1-17 g/ml it is thought to be the GPLV-associated reverse transcriptase. The soluble DNA polymerase is stimulated by ribonuclease and is probably of cellular origin.


Br. J. Cancer (1976) 33, 419

### DNA POLYMERASE ACTIVITY IN PLASMA FROM LEUKAEMIC GUINEA-PIGS

F. M. HALLINAN AND N. MACLEAN

*From the Department of Biology, Medical and Biological Sciences Building, University of Southampton, Bassett Crescent East, Southampton, SO9 3TU*

Received 29 October 1975 Accepted 29 December 1975

**Summary.—Measurement of DNA polymerase in leukaemic guinea-pig plasma reveals the presence of low levels of sedimentable and non-sedimentable enzymic activities. Since the sedimentable DNA polymerase is ribonuclease sensitive, uses poly(C).oligo(dG) as template, and bands in a sucrose density gradient at 1·17 g/ml it is thought to be the GPLV-associated reverse transcriptase. The soluble DNA polymerase is stimulated by ribonuclease and is probably of cellular origin.**

AN RNA-DIRECTED DNA polymerase or reverse transcriptase enzyme has now been shown to be present in many of the RNA tumour viruses and to be sometimes involved in the generation of the neoplastic state (Green and Gerard, 1974).

The guinea-pig L2C leukaemia, an acute lymphoblastoid disease, can be serially transmitted in the inbred strain 2 or hybrid F1 guinea-pigs by injection of whole blood or a spleen tissue suspension from leukaemic animals (Gross *et al.*, 1970). Particles resembling RNA tumour virus virions, termed guinea-pig leukaemia virus (GPLV), have been observed in the tissues of leukaemic animals (Gross *et al.*, 1970; Joachim and Berwick, 1970; Nadel *et al.*, 1967; Opler, 1967) and detected in high-speed plasma pellets (Opler, 1967). Despite earlier reports to the contrary (Jungeblut and Kodza, 1960) it does not appear to be possible to transmit the disease by means of cell-free extracts (Sarma *et al.*, 1970).

Leukaemic guinea-pig plasma has been reported to contain a non-sedimentable DNA polymerase activity (Nayak and Shoyab, 1973) but GPLV-containing plasma pellets although apparently having low levels of DNA polymerase (Nayak and Murray, 1973) have not been systematically examined for reverse transcriptase activity. We have investigated this situation further since the presence or absence of reverse transcriptase from GPLV particles might be of importance in relation to their lack of infectivity. We are here reporting our findings of low levels of both sedimentable and non-sedimentable DNA polymerases in leukaemic guinea-pig plasma.

### MATERIALS AND METHODS

Leukaemic guinea-pig plasma, taken from animals in the terminal stages of the disease, was generously provided by Professor G. Stevenson of the Tenovus Research Laboratory, Southampton General Hospital, U.K. Normal, non-leukaemic guinea-pig blood was obtained by cardiac puncture of strain 2 guinea-pigs. Cells were removed by centrifugation, at 300 *g* for 10 min and the supernatant plasma stored until use. Chick plasma, containing BAI strain Avian Myeloblastosis $Virus_2$ ($AMV_2$) was a kind gift of Dr J. W. Beard, Duke University, U.S.A.

Tritiated deoxynucleoside triphosphates were obtained from the Radiochemical Centre, Amersham, U.K. Poly(C).$(dG)_{12-18}$ was obtained from P–L Biochemicals Inc., Milwaukee, U.S.A. All other synthetic templates, calf thymus DNA, bovine pancreatic ribonuclease and deoxyribonuclease (electrophoretically purified) were from The Sigma Chemical Co. Ltd., London, U.K. All other reagents were Analar grade from British Drug Houses Ltd., Poole, U.K.

*Virus purification.*—Virus from plasma was purified by 3 cycles of low and high speed centrifugation, essentially as described by Bonar *et al.* (1967). As a final step, the virus suspension was centrifuged at 95,000 *g* for 70 min through 20% glycerol in 0·1 M Tris-HCl pH 7·4, 0·05 M NaCl, 0·01 M EDTA (TNE pH 7·4) on to a pad of glycerol as described by Kacian *et al.* (1971). The material on the glycerol pad was then pelleted at 95,000 *g* for 35 min, suspended in 0·05 M Tris-HCl pH 8·0, 0·06 M NaCl (RT buffer) or TNE pH 7·4 (as appropriate) and stored at −20°C. For isopycnic banding of virus particles, aliquots of the glycerol pad pellet in TNE pH 7·4 were layered over pre-cooled 6·0 ml linear gradients formed from 33% and 68% (w/v at 20°C) sucrose solutions in TNE pH 7·4. The tubes were centrifuged for 3 h at 300,000 *g* in an MSE 3 × 6·5 ml Titanium Rotor to achieve equilibrium. The gradients were fractionated into 0·3 ml fractions, by withdrawal from the bottom of the tube through a narrow metal needle connected in series to an LKB 12,000 varioperpex peristaltic pump and an LKB 7000 ultrarac fraction collector, which were stored at −20°C for later analysis.

Samples of the first high speed supernatant and first high speed pellet, resuspended in RT Buffer, were also kept for analysis of the yield and degree of sedimentation of the DNA polymerase activity.

*DNA polymerase assay.*—DNA polymerase activity was measured by mixing equal volumes of solutions A and B with 2 volumes of solution C, and incubating at 37°C. Samples were taken at various times on to Whatman 3MM filter discs which were first washed in 10% trichloroacetic acid containing 50 mM sodium pyrophosphate and then given a series of washes in trichloroacetic acid, essentially as described by Mans and Novelli (1961). The dried discs were counted in PPO–POPOP scintillation fluid in a Packard Tri-Carb liquid scintillation counter.

Solution A contained 50 mM Tris-HCl pH 8·0, 60 mM NaCl, 0·8% Nonidet P-40, 8 mM dithiothreitol, 24 mM magnesium acetate, 8·3 $\mu$M (8-$^3$H) dGTP (15 Ci/mmol) (with poly(C).oligo(dG) as template) *or* 2·5 $\mu$M (Methyl-$^3$H) TTP (50 Ci/mmol), 0·10 mM dATP, 0·10 mM dGTP, 0·10 mM dCTP (with all other templates).

Solution B contained 0·962 $A^{260}$ units/ml of synthetic template *or* 200 $\mu$g/ml of heat denatured calf thymus DNA in RT Buffer.

Solution C contained 0·4–30 mg/ml of plasma protein in RT Buffer.

*Protein and DNA determination.*—Protein concentration was determined, with bovine serum albumin, fraction V as a standard, by a modification of Lowry's method as described in Shatkin (1969). DNA was determined by the diphenylamine method using calf thymus DNA as standard (Shatkin, 1969).

### RESULTS

### 1. *Activity of normal and leukaemic plasma pellets*

In initial experiments the level of incorporation of ($^3$H)-dTTP obtained with high speed plasma pellets, containing AMV or GPLV, in response to the template poly(A).oligo(dT) was examined. The GPLV preparations showed a low (relative to AMV) but significant incorporation of radioactive deoxynucleoside triphosphate.

This incorporation is believed to represent a genuine enzymic activity and not to result from non-specific binding of isotope by the discs, since it is sensitive to heat pre-incubation of the viral material and to 0°C incubation of the reaction mixture (Table I). Further evidence of the enzymic nature of this activity is the near linear plot of protein concentration *vs.* incorporation (Fig. 1).

Further experiments in which a series of leukaemic plasma pellets were analysed, revealed that the amount of DNA polymerase activity varied between samples but was in almost all cases significantly higher than the background level of incorporation obtained with plasma from normal guinea-pigs (Table II).

Examination of the time-course of activity of this enzyme with different templates shows that it exhibits some unusual properties (Fig. 2): (a) the ratio of activities found with poly(A).oligo(dT) relative to poly(dA).oligo(dT) is ap-

TABLE I.—*Characteristics of Endogenous DNA Polymerase Activity*

| Sample | % Incorporation | |
|---|---|---|
| | High-speed supernatant | High-speed pellet |
| Control | 100 | 100 |
| +RNase | 112 | 68 |
| +DNase | 20 | 10 |
| Heat-inactivated protein | 21 | 8 |
| 0°C Incubation | ND* | 21 |
| A-dATP | 70 | 50 |
| A-dGTP | 68 | 53 |

* Not determined.

DNA polymerase activity was measured at 37°C in the absence of added exogenous template. The RNase-treated samples contain RNase at a concentration of 125 μg/ml (pre-heated at 100°C for 10 min to inactivate contaminating DNase). The DNase-treated samples contained EP DNase at a concentration of 25 μg/ml. The heat-inactivated sample contained plasma protein that had been pre-heated at 100°C for 15 min. The A-dATP and A-dGTP samples contained solution A without the specified deoxynucleoside triphosphate.

FIG. 1.—Response of pellet DNA polymerase activity to increasing protein concentration. DNA polymerase activity per 0·1 ml of reaction mixture, in response to poly(A).oligo(dT) was measured in a 30 min incubation at 37°C with different amounts of protein, as described in *Materials and Methods* except that Solution A contained only one deoxyribonucleoside triphosphate, (³H)-dTTP.

proximately 2, which is much less than that shown by the reverse transcriptases of most other RNA tumour viruses (Sarin, Abrell and Gallo, 1974); (b) the ratio of activities with exogenous templates relative to the endogenous template is also less than that found with

TABLE II.—*DNA Polymerase Activity of Different Leukaemic and Control Samples*

| Sample | pmol (³H)-dTMP/mg protein/120 min incubation | |
|---|---|---|
| | High-speed supernatant | High-speed pellet |
| 1 | 1·75 | 18·32 |
| 2 | 1·72 | 16·47 |
| 3 | 2·39 | 76·66 |
| 4 | 2·00 | 14·56 |
| 5 | 1·44 | 18·53 |
| 6 | 3·17 | 62·25 |
| 7 | 0·78 | 5·44 |
| 8 | 1·16 | 9·21 |
| C* | 0·35 | 6·4 |

* A control plasma sample from normal non-leukaemic guinea-pig.

DNA polymerase activity was measured at 37°C with poly(A).oligo(dT) as template. 25 μl samples were taken for determination of TCA precipitable radioactivity, as described in *Materials and Methods.*

FIG. 2.—Time course of pellet DNA polymerase activity with different templates. Reactions were carried out as described in *Materials and Methods* with the following templates: ▲—▲—▲ poly(A).oligo(dT); ■—■—■ poly(dA).oligo(dT); ●—●—● no added template, *i.e.* endogenous.

other RNA tumour virus reverse transcriptases (Spiegelman *et al.*, 1970).

2. *Evidence that the pellet DNA polymerase is a reverse transcriptase*

The demonstration that the endogenous activity of a DNA polymerase is ribonuclease sensitive, although not completely unequivocal (Reitz *et al.*, 1974) provides strong evidence that the enzyme may be a reverse transcriptase. Table I demonstrates that the plasma pellet DNA polymerase activity is reduced both by RNase and DNase treatment of the reaction mixture. The level of incorporation achieved with the DNase treated reaction mixture is approximately the same as that found in a reaction containing heat-inactivated enzyme and is therefore due to non-specific binding. RNase treatment of the reaction mixture reduces the incorporation by 30%. The failure to obtain a complete reduction to the level of the DNase-treated reaction may be due to the presence of DNA-directed DNA polymerase in the preparation or to failure of the RNase to completely digest the endogenous RNA. The former possibility appears the most likely since neither pre-incubation of the enzyme preparation with RNase nor increasing the RNase concentration affected the degree of reduction obtained.

DNA polymerase activity templated by poly(C).oligo(dG) has been postulated to be a specific test for reverse transcriptase (Baltimore, McCaffrey & Smoler, 1973). As seen in Table III the leukaemic plasma pellet will copy this template, indicating that at least some of the DNA polymerase activity of the plasma pellet is due to a virus-associated reverse transcriptase.

Furthermore, in one experiment where sufficient active material was available, the plasma pellet preparation was subjected to isopycnic banding in a sucrose density gradient. The RNase-sensitive DNA polymerase and the poly(C).oligo(dG) DNA polymerase cosedimented at 1·17 g/ml (Fig. 3), the density characteristic of C-type virus particles (Green and Gerard, 1974). These results provide a further, stronger, indication that the plasma pellet DNA polymerase is in fact a virus-associated reverse transcriptase.

TABLE III.—*DNA Polymerase Activity of Leukaemic Plasma Fractions with Poly(C).oligo(dG)*

| | pmol ($^3$H)-dGMP/mg protein | |
|---|---|---|
| Sample | Incorporation at 0°C | Incorporation at 37°C |
| * Supernatant | 0 | 0 |
| † Pellet | 0 | 19·8 |

* The supernatant after one spin at 95,000 *g* for 35 min.

† The pellet obtained after one spin at 95,000 *g* for 35 min.

DNA polymerase activity in response to the template poly(C).oligo(dG) was measured at 0°C (as control) and 37°C in a 120 min incubation.

3. *DNA Polymerase activity of the high speed supernatant*

The plasma of leukaemic guinea-pigs, unlike that from other species, has been reported to possess a non-sedimentable DNA-dependent DNA polymerase (Nayak and Shoyab, 1973). We have studied this activity and its relationship to the DNA polymerase activity in GPLV containing pellets. The results of an analysis of the DNA polymerase activities of the different fractions obtained during the purification of AMV from chick plasma and GPLV from guinea-pig plasma are presented in Table IV. These results illustrate the increased purification and higher specific activity of AMV reverse transcriptase relative to the GPLV polymerase. More significant, however, is the fact that the percentage of the DNA polymerase activity that is non-sedimentable is approximately 0·07% for chick plasma but is 7% for guinea-pig plasma. This incorporation represents a genuine DNA polymerase since, as shown in Table I, it is sensitive to heat pre-incubation of the presumptive enzyme and to

FIG. 3.—Sucrose equilibrium density gradient centrifugation of the pellet DNA polymerase. An aliquot of the glycerol pad pellet was subject to sucrose density gradient centrifugation as described in *Materials and Methods.* DNA polymerase activity of the different gradient fractions was measured in a 60 min incubation at 37°C, with poly(C).oligo(dG): ★—★—★, in an endogenous reaction: ▲—▲—▲, and in an RNase treated (125 μg/ml) endogenous reaction: ■—■—■. For the endogenous reactions aliquots of each gradient fraction were pre-incubated at 37°C for 15 min with or without added RNase.

TABLE IV.—*DNA Polymerase Activity of Different Fractions from Chick and Guinea-pig Plasma*

| Sample | pmol ($^3$H)-dTMP/mg protein: Chick plasma | Guinea-pig plasma |
|---|---|---|
| Low-speed supernatant | 14·0 | 5·9 |
| High-speed supernatant | 3·2 | 3·8 |
| First pellet | 3817·7 | 73·0 |
| Final pellet | 20255·3 | 104·3 |

DNA polymerase activity was measured in a 60 min incubation with poly(A).oligo(dT) as template, as described in Materials and Methods. Low-speed supernatant is the supernatant after centrifugation at 2000 *g* for 10 min to remove cellular debris. High-speed supernatant and first pellet are obtained after one spin at 95,000 *g* for 35 min. Final pellet is obtained after centrifugation through 20% glycerol in TNE on to a glycerol pad.

omission of deoxynucleoside triphosphates from the reaction mixture. The amount of DNA polymerase activity in different leukaemic supernatant samples, like that in the pellet, varies widely, as shown in Table II, and there does not appear to be any simple relationship between the levels of the supernatant and pellet enzymes.

These results are in agreement with those of Nayak and Shoyab (1973) and imply, either that there are two different DNA polymerases in leukaemic guinea-pig plasma, or that the GPLV virions are somehow " leaky " and release their DNA polymerase into the medium.

In an attempt to distinguish between these two hypotheses we examined the template specificities of the polymerase activities in the high speed supernatant and pellet as shown in Table V. The two enzymes have different specific activities due to different degrees of purification. Denatured DNA is a poor template for both enzymes since their activities are reduced relative to the endogenous level.

TABLE V.—*DNA Polymerase Activity of Leukaemia Guinea-pig Plasma Pellet and Supernatant with Different Templates*

| | pmol ($^3$H)-dTMP/mg protein | | | |
|---|---|---|---|---|
| Sample | Poly(A).oligo(dT) | Poly(dA).oligo(dT) | Denatured DNA | Endogenous |
| Supernatant | 7·66 | 7·02 | 3·1 | 7·65 |
| Pellet | 48·3 | 32·5 | 21·3 | 36·6 |

DNA polymerase activity was measured in a 60 min incubation with the different templates shown. The supernatant is the high-speed supernatant. The pellet was obtained after glycerol pad centrifugation.

However, the supernatant activity, unlike that of the pellet, is not stimulated by poly(A).oligo(dT). In addition, the supernatant activity differs from the pellet in being stimulated by RNase (Table I), and in being unable to copy poly(C).oligo(dG) (Table II). The lack of any soluble DNA polymerase in the supernatant after further high speed centrifugation illustrates, at least, that DNA polymerase activity is not being continuously lost by the GPLV-containing pellet material.

Therefore it seems likely that these two enzymes have a different origin. In fact, a partially purified DNA polymerase from leukaemic cell nuclei shows an activation of its endogenous activity by RNase treatment (Hallinan and Maclean, unpublished) as does the supernatant DNA polymerase.

### DISCUSSION

The two possible origins of the leukaemic plasma DNA polymerases described here are cellular or viral. Both normal and leukaemic cells contain predominantly three cellular DNA polymerases (reviewed by Loeb, 1975; Bollum, 1975). Although dissimilar from the major cytoplasmic and nuclear enzymes (DNA polymerase $\alpha$ and $\beta$) which have a preference for DNA templates, the leukaemic pellet enzyme resembles the DNA polymerase $\gamma$ or III (the reverse transcriptase-like cellular enzyme (Lewis *et al.*, 1974)) in displaying a preference for poly(A).oligo(dT). The leukaemic pellet enzyme however, unlike DNA polymerase $\gamma$, can copy poly(C).oligo(dG), a property which appears to be confined to the RNA tumour virus reverse transcriptase (Baltimore *et al.*, 1973). Neither have there been any reports, to our knowledge, that DNA polymerase $\gamma$ in crude extracts exhibits RNase sensitivity or is associated with particles of the density of C-type virions. The partial RNase sensitivity of the pellet DNA polymerase was surprising since other reverse transcriptases which have been examined exhibit an 80–90% inhibition at such RNase concentrations (Green and Gerard, 1974). This degree of inhibition was reproducible however, and was not increased by density-gradient sedimentation of the particle-associated enzyme (Fig. 3), implying that some contaminating DNA or DNA-directed DNA polymerase may be present. Nevertheless this partial RNase sensitivity does indicate that at least some of the DNA polymerase activity is RNA directed. The properties of the leukaemic pellet enzyme therefore are unlike those of normal cellular DNA polymerases and it seems likely that this enzyme is viral in origin.

Unequivocal proof that a DNA polymerase is in fact a reverse transcriptase requires a demonstration that the enzyme will copy heteropolymeric regions of natural RNA (Green and Gerard, 1974). However, when dealing with extracellular presumptive viral material as opposed to cellular fractions, other characteristics of the enzyme such as template specificity, ribonuclease sensitivity of the endogenous reaction and density of the enzyme-containing particulate element are useful and permit a preliminary diagnosis of the type of DNA polymerase involved.

Thus, the properties of the pellet DNA polymerase, such as preference for poly(A).oligo(dT), utilization of poly(C).oligo(dG), RNase sensitivity, and association with a particulate element of 1·17 g/ml density, taken in conjunction, indicate that this enzyme is probably a virus-specific reverse transcriptase.

The presence of a soluble, RNase insensitive, DNA polymerase in the leukaemic plasma of these animals, as reported by Nayak and Shoyab (1973), is confirmed by our results. Although this enzyme, like the pellet DNA polymerase, copies poly(A).oligo(dT) better than denatured DNA, it cannot copy poly(C).oligo(dG) and is stimulated by RNase. The inability to utilize poly(C).oligo(dG) as template could possibly be due to the degradation of this template by nucleases in the relatively crude plasma supernatant, since it is reported to be especially sensitive to degradative enzymes (Sarin and Gallo, 1974). However, it is difficult to imagine how the enzyme, if it is of viral origin, could change from being inhibited by RNase to being stimulated on solubilization. Additionally, the partial purification of a nuclear DNA polymerase from these cells, the endogenous activity of which is also stimulated by RNase (Hallinan & Maclean; unpublished) makes it more likely that the soluble DNA polymerase is cellular in origin.

The L2C leukaemia mimics its human counterpart more closely than any other animal leukaemia (Opler, 1969) and studies of the activity of the DNA polymerases in L2C plasma may well be useful as a model system in a search for RNA tumour virus information in human leukaemic plasma.

This research was supported by a grant from The Tenovus Organization.

We are grateful to Professor G. Stevenson of the Tenovus Laboratory, Southampton General Hospital, England, for the generous provision of plasma from leukaemic guinea pigs.

REFERENCES

Baltimore, D., McCaffrey, R. & Smoler, D. F. (1973) Properties of Reverse Transcriptases in Virus Research: in *Second ICN UCLA Symposium on Molecular Biology*. Ed. C. F. Fox & W. S. Robinson. New York: Academic Press.

Bollum, F. J. (1975) Mammalian DNA Polymerases. *Prog. nucl. Acid Res. mol. Biol.*, **15**, 109.

Bonar, R. A., Sverak, L., Bolognesi, D. P., Langlois, A. J., Beard, D. & Beard, J. W. (1967) Ribonucleic Acid Components of BAT Strain A (Myeloblastosis) Avian Tumor Virus. *Cancer Res.*, **27**, 1138.

Green, M. & Gerard, G. F. (1974) RNA Directed DNA Polymerase-Properties and Functions in Oncogenic RNA Viruses and Cells. *Prog. nucl. Acid Res. mol. Biol.*, **14**, 187.

Gross, L., Dreyfuss, Y., Ehrenreich, T. & Moore, L. A. (1970) Experimental Studies on Leukemia in Guinea Pigs. *Acta haematol.*, **43**, 193.

Joachim, H. L. & Berwick, L. (1970) Leukemia of Guinea Pigs. *Biblthca haemat.*, **36**, 556.

Jungeblut, C. W. & Kodza, H. (1960) Attempts to Adapt Leukemia L2C from Inbred to Non-inbred Guinea Pigs. *Am. J. Path.*, **37**, 191.

Kacian, D. L., Watson, K. F., Burny, A. & Spiegelman, S. (1971) Purification of the DNA Polymerase of Avian Myeloblastosis Virus. *Biochim. Biophys. Acta*, **246**, 365.

Lewis, B. J., Abrell, J. W., Smith, R. G. & Gallo, R. C. (1974) DNA Polymerases in Human Lymphoblastoid Cells Infected with Simian Sarcoma Virus. *Biochim. Biophys. Acta*, **349**, 148.

Loeb, L. A. (1975) Eucaryotic DNA Polymerases. In *The Enzymes*, Vol. 10. Ed. P. D. Boyer. New York: Academic Press.

Mans, R. J. & Novelli, G. D. (1961) Measurement of the Incorporation of Radioactive Amino Acids into Proteins by a Filter Paper Disc Method. *Archs Biochem. Biophys.*, **94**, 48.

Nadel, E. M., Banfield, W., Burstein, S. & Tousimis, A. J. (1967) Virus Particles Associated with Strain 2 Guinea Pig Leukemia. *J. natn. Cancer Inst.*, **38**, 979.

Nayak, D. P. & Murray, P. R. (1973) Induction of Type C Viruses in Cultured Guinea Pig Cells. *J. Virol.*, **12**, 177.

Nayak, D. P. & Shoyab, M. (1973) Soluble Plasma DNA Polymerase and Guinea Pig Leukemia. *Life Sciences*, **13**, 575.

Opler, S. R. (1967) Observation of a New Virus Associated with Guinea Pig Leukemia: Preliminary Note. *J. natn. Cancer Inst.*, **38**, 797.

Opler, S. R. (1969) Morphology of Cavian Leukemia. *Natn. Cancer Inst. Monogr.*, **32**, 65.

Reitz, M. S., Smith, R. G., Roseberry, E. A. & Gallo, R. C. (1974) DNA-Directed and RNA-Primed DNA Synthesis in Microsomal and Mitochondrial Fractions of Normal Human Lymphocytes. *Biochem. biophys. Res. Commun.*, **57**, 934.

Sarin, P. S., Abrell, J. W. & Gallo, R. C. (1974) Comparison of Biochemical Characteristics of Reverse Transcriptase from Human Acute Leukemic Cells and Several RNA Tumor Viruses. In *Control of Transcription*. Ed. B. B. Biswas and R. K. Mandall. New York: Plenum Press.

SARIN, P. S. & GALLO, R. C. (1974) RNA Directed DNA Polymerase. In *MTP International Review of Science*, Vol. 6. *Biochemistry of Nucleic Acids*. Ed. K. Burton. London: Butterworth.

SARMA, P. S., UEBERHORST, P. J., ZEVE, V., WHANG-PENG, J. & HUEBNER, R. (1970) L2C/N-B Guinea Pig Leukemia: Failure to Demonstrate Transmissible Leukemogenic Virus. *Biblthca haemat.*, **36**, 574.

SHATKIN, A. J. (1969) Color Tests for DNA, RNA and Protein. In *Fundamental Techniques in Virology*. Ed. K. Habel & N. P. Salzman. New York: Academic Press.

SPIEGELMAN, S., BURNY, A., DAS, M. R., KEYDAR, J., SCHLOM, J., TRAVNICEK, M. & WATSON, K. (1970) Synthetic DNA-RNA Hybrids and RNA-RNA Duplexes as Templates for the Polymerases of the Oncogenic RNA Viruses. *Nature, Lond.*, **228**, 430.